# Seroprevalence, Isolation, Genotyping, and Pathogenicity of *Toxoplasma gondii* Strains from Sheep in China

**DOI:** 10.3389/fmicb.2017.00136

**Published:** 2017-02-03

**Authors:** YuRong Yang, YongJie Feng, QiuXia Yao, YingHua Wang, YaoYao Lu, HongDe Liang, XingQuan Zhu, LongXian Zhang

**Affiliations:** ^1^College of Animal Science and Veterinary Medicine, Henan Agricultural UniversityZhengzhou, China; ^2^State Key Laboratory of Veterinary Etiological Biology, Key Laboratory of Veterinary Parasitology of Gansu Province, Lanzhou Veterinary Research Institute, Chinese Academy of Agricultural SciencesLanzhou, China; ^3^Center for Animal Disease Control and Prevention of Henan ProvinceZhengzhou, China

**Keywords:** *Toxoplasma gondii*, seroepidemiology, isolation, genotype, virulence, sheep, China

## Abstract

*Toxoplasma gondii* is an important cause of reproductive failure in small ruminants that also poses a risk to consumers who consume undercooked meat. However, little is known about sheep toxoplasmosis in China for the world. Therefore, this study was conducted to assess the prevalence of *T. gondii* infection in sheep from China, to isolate *T. gondii* via bioassay in mice and to evaluate the virulence of the isolated *T. gondii* based on vero cell invasion and mice. A total of 840 samples (304 unfrozen hearts and 536 sera) from sheep in China were collected from 2014 to 2016. Heart samples (*n* = 36) of *T. gondii* seropositive sheep (MAT, ≥25) were bioassayed in mice individually. DNA derived from cell cultured tachyzoites of the isolated *T. gondii* was characterized by PCR-RFLP of 10 loci (SAG1, SAG2, SAG3, BTUB, GRA6, c22-8, c29-2, L358, PK1, and Apico). The virulence of the *T. gondii* was evaluated based on the mortality and encystation in mice, as well as their growth characteristics in cell culture. Antibodies to *T. gondii* were found in 174 of 840 (20.71%, 304 hearts juice and 536 sera) sheep by the modified agglutination test (cut-off 1:25). Viable *T. gondii* was isolated from the hearts of two of 36 seropositive sheep hearts. Both genotypes of the sheep heart isolates were ToxoDB#9. The virulence of the two ToxoDB#9 isolations varied significantly. To the best of our knowledge, this is the first report of isolation of ToxoDB#9 strain of *T. gondii* from sheep in China.

## Introduction

The parasite *Toxoplasma gondii* is a major cause of reproductive failure in small ruminants, including sheep. Veterinary Investigation Diagnosis Analysis data from 2014 showed that about 25% of ovine production problems were caused by *T. gondii* (www.gov.uk/government/statistics). Moreover, viable *T. gondii* has been isolated from goat meat, milk and cheese (Dubey et al., 2014a,b). *T. gondii* infection is widespread among humans. The prevalence of *T. gondii* is higher in Latin America than in North America and East Asia (Dubey, 2010); however, the cause of this difference is not known. *T. gondii* cause lymphadenopathy, retinochoroiditis, encephalitis, abortion, and the death of immunocompromised patients (Hide, 2016). In China, mutton is the main ingredient of hotpot, which often results in meat being undercooked. Therefore, the consumption of undercooked meat containing *T. gondii* tissue cysts could pose a health risk to consumers.

Isolation of viable *T. gondii* from feline has been the most successful model in China. Among 122 viable *T. gondii* isolates from animals and humans in China, 85 strains (69.7%) were isolated from tissue or fecal samples from cats. Moreover, 73 (85.9%) *T. gondii* isolates from cats were genotyped as ToxoDB#9, while 10 were genotyped as ToxoDB#1(1), ToxoDB#2(1), ToxoDB#10(1), ToxoDB#17(1), ToxoDB#18(2), and ToxoDB#205(4), and the genotypes of the other two strains were not determined (Dubey et al., 2007; Zhou et al., 2009; Chen et al., 2011; Qian et al., 2012; Wang et al., 2013a; Li et al., 2015; Yang et al., 2015; Wang D. et al., 2016; Wang Q. Q. et al., 2016). However, only one isolate of *T. gondii* has been obtained from sheep in Qinghai (type II) (Zhou et al., 2009), and no studies have reported isolation of viable *T. gondii* from other small ruminants in China.

China has the largest number of sheep in the world, with an estimated 187 million domestic sheep (https://top5ofanything.com/list/d4d1ef5e/Countries-With-the-Most-Sheep) and an unknown number of wild sheep. The clinical and economic importance of sheep toxoplasmosis remains uncertain, and most epidemiological literature was published in Chinese. Therefore, the present study was conducted to summarize these Chinese papers and present the sero-prevalence in sheep from different geographical areas. Further, the prevalence of *T. gondii* infections in sheep from China was investigated, and an attempt to isolate viable *T. gondii* was made.

## Materials and methods

### Sheep sample collection

**Figure 1:**
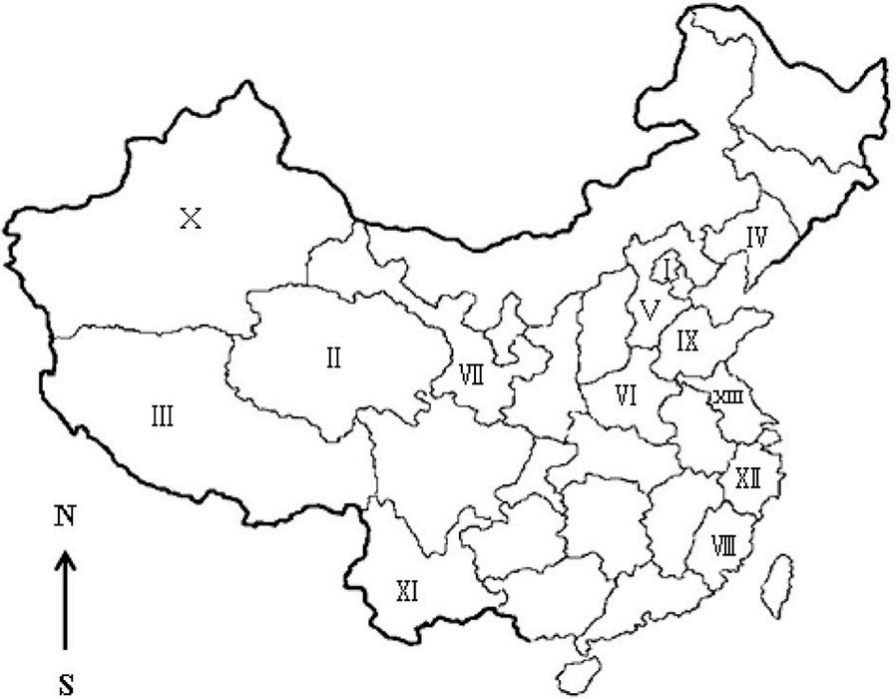
**Seroepidemiology of ***Toxoplasma gondii*** in sheep in China**. I, Beijing; II, Qinghai; III, Tibet; IV, Liaoning; V, Hebei; VI, Henan; VII, Gansu; VIII, Fujian; IX, Shandong; X, Xinjiang; XI, Yunnan; XII, Zhejiang; XIII, Jiangsu.

A total of 840 domestic sheep samples (304 fresh hearts and 536 sera) were collected from individual farms in Henan, Xinjiang, Zhejiang, and Jiangsu Province (Table 1, Figure 1). The climate of Henan Province (33°N, 113.30°E) is humid and subtropical, whereas Zhejiang Province (29.12°N, 120.30°E) is characterized by a subtropical monsoon climate. Jiangsu Province (Latitude 32.54°N, Longitude 119.48°E) is situated in the climatic transition belt between the warm-temperate and subtropical zones. Xinjiang (41°N, 85°E) has a semi-arid and desert climate. Domestic sheep in China are part of the farmers's household, and live with whatever other animals the farmers own, including cats. Unfrozen hearts and serum from sheep were collected between 2014 and 2016. In addition, juice was obtained from 304 hearts. Blood was obtained from jugular veins of 536 sheep. The juice or blood samples were allowed to clot, then centrifuged at 2000 × g for 10 min, after which the supernatant were separated and stored at −20°C until tested. Available background information is summarized in Table 1.

**Table 1:** **Seroprevalence and isolation of ***Toxoplasma gondii*** in sheep**.

**Batch number**	**Location^a^**	**Province**	**City**	**Sample received date**	**No. of Samples^b^**	**% (Positive No./Test No.) cut-off titer:1:25**	**Isolation obtained by mice from sample^c^**
1	VI	Henan (*n* = 283)	Hebi	16 Oct 2014	67 Sera	29.33 (83/28)	–
2				10 May 2015	8 Sera		–
3				12 May 2015	6 Sera		–
4				23 June 2015	2 Hearts		0/1
5			Xinyang	24 May 2015	1 Heart		0/1
6				2 June 2015	1 Heart		0/1
7				9 June 2015	1 Heart		–
8				8 July 2015	4 Hearts		–
9			Xuchang	29 May 2015	5 Hearts		–
10				23 June 2015	4 Hearts		–
11				2 June 2016	5 Hearts		–
12			Zhumadian	8 Oct 2015	15 Hearts		0/1
13				30 Nov 2015	31 Hearts		0/2
14				1 Dec 2015	20 Hearts		–
15				8 Dec 2015	20 Hearts		0/7
16			Luoyang	19 Oct 2015	23 Hearts		0/14
17			Jiaozuo	10 Nov 2015	70 Hearts		2/7
18	XII	Zhejiang (*n* = 208)		30 Oct 2014	208 Sera	21.15 (44/208)	–
19	XIII	Jiangsu (*n* = 247)		6 Dec 2014	247 Sera	18.22 (45/247)	–
20	X	Xinjiang (*n* = 102)		5 Jan 2016	102 Hearts	1.96 (2/102)	0/2
Total					304 Hearts	20.71	2/36
					536 Sera	(174/840)	

a *Sampling province in Figure 1*.

b *One heart sample and one serum sample were collected from the same sheep*.

c *Number of positive groups/number of inoculated groups*.

### Serological examination

Serum and heart juice samples from 840 sheep were tested for antibodies to *T. gondii* using the modified agglutination test (MAT) (Dubey and Desmonts, 1987). Whole formalin fixed RH *T. gondii* tachyzoites were kindly provided by Dr. J. P. Dubey (ARS, USDA). A titer of 1:25 was considered indicative of exposure to *T. gondii*. In addition, sera and heart juice were double diluted further with 0.01 M phosphate buffered saline (PBS), then tested for *T. gondii* parasites. Briefly, 100 mL 0.01 M PBS was amended with 8.5 g NaCl, 0.308 g NaH_2_PO_4_ (M.W. 120), and 1.08 g Na_2_HPO_4_ (M.W. 142), after which the pH was adjusted to 7.2 (Dubey, 2010).

### Isolation of viable *T. gondii* from sheep hearts by bioassay in mice

Specific-pathogen-free *Kunming* mice were supplied by the Zhengzhou University Laboratory Animal Center. Eight-week-old female *Kunming* mice were used in this study. Heart samples of *T. gondii* seropositive sheep (MAT, ≥25) were bioassayed in mice separately. The myocardium (50 g) was then homogenized, digested in pepsin (5.2 g pepsin, 10.0 g NaCl, 14 mL HCl, diluted to 1 l with deionized water, pH 1.1–1.2). The heart homogenate was subsequently incubated at 37°C in a shaking water bath for 60 min. After which, the sample was filtered by double gauze and centrifuged at 1200 × g for 10 min. The supernatant was then removed and the pellet was suspended in 0.01 M PBS (pH 7.2) and neutralized by mixing with 1.2% sodium bicarbonate. Following mixing, the sample was centrifuged at 1200 × g for 10 min, after which the supernatant was removed and 5–10 mL of saline containing 1000 units penicillin and 100 μg of streptomycin per ml was added. Myocardium digested liquid was then inoculated subcutaneously into four *Kunming* mice (1 mL per mouse) that had been maintained on drinking water supplemented with dexamethasone phosphate (DXM, 10 μg/ml) for 3 days before inoculation (Dubey, 2010). DXM treated mice were utilized as a control group, while DXM untreated mice were utilized as a blank group. Lung or brain impression smears of dead mice were examined for *T. gondii* tachyzoites or cysts. Survivors were bled on day 60 post-inoculation (DPI) and 120 DPI. 1:25 and 1:200 dilutions of sera from each mouse were tested for *T. gondii* antibodies with the MAT. Mice were killed 120 DPI and their brains were examined for tissue cysts after a squash preparation. All brains of survivors were homogenized and sub-passaged into new groups of mice subcutaneously.

### *In vitro* cultivation and genotyping

Brain homogenates of *T. gondii* positive mice were seeded into vero cell culture flasks as previously described (Dubey, 2010). The number of cysts in the brains of mice were counted microscopically using the method reported by Dubey et al. (2012). Briefly, whole mouse brain was homogenized with 1 mL of saline (0.85% NaCl), tissue cysts were counted microscopically in 50 μl of the homogenate, and the count multiplied by 20 was the number of tissue cysts per brain. The time required for tachyzoites grow up in cell culture was recorded. DNA was extracted from cell culture derived tachyzoites using a commercial DNA extraction kit (Tiangen Biotec Company, DP304, China). The multiplex PCR of the *T. gondii* isolates was performed using 10 PCR-RFLP genetic markers, SAG1, SAG2 (5′–3′ SAG2, alt.SAG2), SAG3, BTUB, GRA6, c22-8, c29-2, L358, PK1, and Apico as previously described by Su et al. (2010). Reference *T. gondii* DNA was included in all batches.

### Evaluation the virulence of *T. gondii* tachyzoites isolated from sheep by mice

Fresh tachyzoites were collected from cell culture, counted in a disposable hemocytometer and diluted 10-fold from 10^−1^ to 10^−6^ to reach an end-point of <1 tachyzoite. Next, <1, 10^0^, 10^1^, 10^2^, 10^3^, or 10^4^ tachyzoites were inoculated intraperitoneally into five *Kunming* mice for each dilution. Clinical symptoms, illness and death of mice were observed and recorded every day. Lung or mesenteric lymph node impression smears of dead mice were examined for *T. gondii* tachyzoites. At 30 DPI, sera from mice were analyzed for *T. gondii* antibody. The virulence was evaluated based on the percentage of dead mice among *T. gondii* positive mice.

### Ethics approval and consent to participate

This study was carried out in accordance with the recommendations of the institutional animal use committee of the Henan Agricultural University (China). The protocol was approved by the Beijing Association for Science and Technology (approval SYXK [Beijing] 2007-0023).

### Statistical analysis

Statistical analysis was performed using the Graph Pad Prism 4.0 software (Graph Pad Software Inc., San Diego, CA, USA). Data were analyzed by the chi-squared test or Fisher's exact test. A *P* < 0.05 was considered statistically significant.

## Results

### Seroepidemiology of *T. gondii* in sheep

Antibodies to *T. gondii* were found in 174 of 840 (20.71%) sheep with titers of 1:25 in 48, 1:50 in 2, 1:100 in 24, and 1:200 or above in 100. Seropositivity rates varied with respect to source of sheep. The difference in seroprevalence of *T. gondii* in sheep from Henan Province (29.33%), Zhejiang Province (21.15%) and Jiangsu Province (18.22%) was not significant. However, the prevalence was higher in all of these regions than in Xinjiang (1.96%) (*P* < 0.05) (Table 1).

Detailed information was only available for 522 of the 840 samples. Female sheep (18.64%, 74/397) shows a tendency to be more susceptible to *T. gondii* (odds ratio = 1.278) than male sheep (15.20%, 19/125), but this difference was not significant. The seroprevalence of *T. gondii* in aborting sheep (41.30%, 38/92) was higher than that in non-aborting sheep (11.80%, 36/305), with an odds ratio of 5.258 (95% CI, 3.059–9.038), which was statistically significant (*P* < 0.0001). In this study, 16.22% (6/37) of sheep <1 year old were seropositive for *T. gondii*, while17.93% (87/485) of those >1 year old were seropositive. The risk of acquiring *T. gondii* infection in adult sheep (17.93%) tended to be higher when compared to that in lambs (16.22%), with an odds ratio of 1.129 (95% CI, 0.4571–2.7910); however, this difference was not statistically significant (*P* > 0.05).

### Isolation and virulence of *T. gondii* from sheep

A total of 36 *T. gondii* seropositive sheep heart homogenates were bioassayed in four *Kunming* mice individually. *T. gondii* antibodies were only detected in 10 groups of mice at 60 DPI. Specifically, one mouse was positive in eight groups, three mice were positive in one group, and four mice were positive in one group. However, *T. gondii* antibodies were only detected in mice from the last two groups at 120 DPI, while the other eight groups were negative. Viable *T. gondii* were isolated from the two positive groups by *Kunming* mice (Table 1). All mice remained asymptomatic. *T. gondii* tissue cysts from the brain were detected in these mice when killed at 120 DPI. The average number of brain cysts in *T. gondii* infected mice was 1900 ± 141 from sheep heart sample 20151110#24, while 100 ± 53 cysts were observed in heart sample 20151110#28. The brain homogenates of *T. gondii* positive mice were sub-inoculated into mice, and seeded onto cell cultures for propagation of tachyzoites. Two isolates (TgSpHn1, TgSpHn2) were successfully propagated in cell culture and mice. Genetic typing of the isolates from sheep hearts revealed that they were all ToxoDB genotype #9 (Chinese 1) (Table 2).

**Table 2:** **Isolation of viable ***T. gondii*** from sheep hearts by bioassay in mice**.

**Sample ID**	**Source**	**Ownership**	**Age (days)**	**MAT titer**	**Mice bioassay^b^**	**No. of cysts in mice (120 DPI^c^)**	**Time grow up in cell culture**	**Isolate designation**	**Toxo DB genotype**
20151110#24^a^	Jiaozuo, Henan	Household	250	12800	4/4	1900 ± 141^d^	10 days	TgSheepHn1	#9
20151110#28^a^	Jiaozuo, Henan	Household	250	12800	3/4	100 ± 53^d^	25 days	TgSheepHn2	#9

a *Sex was unknown*.

b *No. of mice infected with T. gondii/No. of mice inoculated*.

c *DPI, days post-inoculation*.

d *The data means average cysts in brain per infected mouse*.

Mice showed 100% mortality after inoculation with 100 *T. gondii* tachyzoites of TgSheepHn1. For TgSheepHn2, the 100% mortality dose was 1000 tachyzoites. However, loading with the highest level of 10^4^ tachyzoites per mouse induced only 80% (TgSheepHn1) and 60% mortality (TgSheepHn2). The survival time post-inoculation with 10^4^, 10^3^, 10^2^, 10^1^, and 10^0^ TgSheepHn1 parasites was 8, 9.5 days, 259 h, 20.5, and 17.5 days, while it was 8, 22 days, 343 h, above 30 days and above 30 days for TgSheepHn2, respectively (Table 3). The survival time post-inoculation with *T. gondii* tachyzoites differed between the two isolates from sheep.

**Table 3:** **Pathogenicity of the two isolated ***T. gondii*** tachyzoite strains with gradient dilution dosage on ***Kunming*** mice inoculated by intraperitoneal injection (30 DPI)**.

**Concentration of tachyzoites**	**10^0^**	**10^1^**	**10^2^**	**10^3^**	**10^4^**
**TgSheepHn1 (ToxoDB#9)**					
*T. gondii* positive rate %	60	100	100	100	100
(No.infected/No. inoculation)	(3/5)	(5/5)	(5/5)	(5/5)	(5/5)
Mortality%	66.7	80	100	80	80
(No. died/No. infected)	(2/3)	(4/5)	(5/5)	(4/5)	(4/5)
Range of survival time	14, 21 days	12–30 days	9–13 days	9–11 days	8 days
(Median survival time)	(17.5 days)	(20.5 days)	(259 h)	(9.5 days)	
**TgSheepHn2 (ToxoDB#9)**					
*T. gondii* positive rate %	40	60	100	100	100
(No. infected/No. inoculation)	(2/5)	(3/5)	(5/5)	(5/5)	(5/5)
Mortality%	0	0	80	100	60
(No. died/No. infected)	(0/2)	(0/3)	(4/5)	(5/5)	(3/5)
Range of survival time	>30 days	>30 days	13–16 days	21–24 days	8 days
(Median survival time)			(343 h)	(22 days)	

## Discussion

In this study, the prevalence of antibodies and titers to *T. gondii* in sheep from Henan, Jiangsu, and Zhejiang province was higher than in sheep from Xinjiang (Table 1). The climate of Henan, Zhejiang, and Jiangsu is subtropical, whereas that of Xinjiang is semi-arid and desert. The arid climate of Xinjiang may contribute to the low prevalence of toxoplasmosis in the region. These results are consistent with those of other reports (Table 4, Figure 1). We have summarized available reports on sheep toxoplasmosis in Table 4. The prevalence of antibodies and titers to *T. gondii* was 2–39% in sheep from different parts of China (Table 4), indicating widespread environmental contamination with *T. gondii* oocysts. These findings are in accordance with those of our previous investigation of *T. gondii* in free-range chickens (Feng et al., 2016).

**Table 4:** **Prevalence of ***Toxoplasma gondii*** antibodies in sheep in the People's Republic of China**.

**Map region^h^**	**Province**	**Year tested**	**Type**	**No. tested**	**No. positive**	**% Positive**	**Serologic test (cut-off titer)**	**References**
I	Beijing	<2006	Domestic	230	90	39.31	PAPS^a^	Wang et al., 2006
II	Qinghai	2008–2010	Tibetan sheep	930	29	3.12	IHA^b^ (1:64)	Li, 2012
		<2009	Tibetan sheep	108	4	3.70	IHA^b^ (1:64)	Zhang et al., 2009a
		2010	Tibetan sheep	580	173	29.80	IHA^c^ (1:64)	Liu et al., 2010
		2006	Domestic	237	10	4.22	IHA^b^ (1:64)	Wang, 2007
		<2011	Domestic	600	174	29.00	IHA^b^ (1:64)	Dong et al., 2011
		<2009	Tibetan sheep	360	10	2.78	IHA^b^ (1:64)	Zhang et al., 2009b
		2007	Domestic	223	6	2.69	IHA^b^ (1:64)	Yuan and Ma, 2007
		<2003	Domestic	180	14	7.78	IHA^b^ (1:64)	Fu, 2003
		<2008	Domestic	56	16	28.60	IHA^b^ (1:64)	Chen, 2008
		2012–2013	Domestic	600	128	21.33	ELISA^d^, IHA (1:50)	Liu et al., 2015
III	Tibet	2011	Tibetan sheep	455	26	5.70	IHA^b^ (1:64)	Wu et al., 2011
IV	Liaoning	2011	Domestic sheep	566	25	4.40	IHA^b^ (1:64)	Yang et al., 2013
V	Hebei	<2004	Domestic	222	57	25.68	IHA^b^ (1:64)	Cui et al., 2004
		<2004	Domestic	128	17	13.28	ELISA^e^	Yuan et al., 2004
VI	Henan	2004	Domestic	50	8	16.00	IHA^b^	Wang et al., 2005
		<2007	domestic	565	119	21.00	IHA^b^ (1:64)	Zhu et al., 2007
		2015–2016	Domestic	779	99	12.71	MAT (1:25)	Zhang et al., 2016
VII	Gansu	2013–2014	Tibetan sheep	1732	352	20.30	MAT^f^ (1:25)	Yin et al., 2015
VIII	Fujian	<2013	Domestic	35	8	22.86	ELISA^g^	Luo et al., 2013
IX	Shandong	<2001	Domestic	276	65	23.55	IHA^b^ (1:64)	Zhao et al., 2001
X	Xinjiang	<2002	Domestic	289	29	10.00	IHA^b^ (1:64)	Bai et al., 2002
		<2009	Domestic	409	23	5.62	IHA^b^ (1:64)	Zhang Y.-f. et al., 2009
		2014	Farm	486	10	2.05	IHA	Wang Q. Q. et al., 2016
XI	Yunnan	<2002	Domestic	258	16	6.20	IHA^b^ (1:64)	Ye et al., 2002

*PAPS, polyaledehyde polystyrene; IHA, indirect hemagglutination test; ELISA, enzymelinked immunosorbent assay*.

a *Shanghai Institute of Parasitic Diseases, China*.

b *Lanzhou Veterinary Institute, Academy of Agriculture and Science, China*.

c *Veterinary Research Institute, Jiangsu Academy of Agricultural Sciences, China*.

d *IDEXX Laboratories*.

e *Zhejiang Institute of Parasitic Diseases, Academy of Agriculture and Science, China*.

f *In-house*.

g *Zhuhai Biological Pharmaceuticals co., Ltd*.

h *Province in Figure 1*.

Oral ingestion of *T. gondii* oocysts is the main source of infection for sheep, and poses a risk for exogenous transplacental transmission in pregnant sheep (Innes et al., 2009). Moreover, a previous study confirmed that reactivation of *T. gondii* cysts in chronically infected sheep serves as another important risk for endogenous transplacental transmission in sheep during pregnancy (Williams et al., 2005; Hide, 2016). Additionally, mutton containing *T. gondii* tissue cysts poses a threat to human health. Mutton is the main ingredient of hot pots, instant-boiled mutton, dumpling and kebabs in China, however, these cooking methods are often not sufficient to eradicate *T. gondii*. Moreover, the high prevalence of *T. gondii* in aborting sheep observed in the present study indicate that *T. gondii* is a risk factor for abortion. Increasing rates of prevalence of *T. gondii* antibodies in older sheep indicated post-natal exposure of *T. gondii* infection, which agreed with the results of previous studies (Dubey, 2010).

*T. gondii* antibodies were detected in mice of 10 groups at 60 DPI, but were no longer present in eight groups at 120 DPI. Moreover, mice in eight groups were all negative for *T. gondii* after sub-passage in mice. After checking the *T. gondii* DNA of the eight injected sheep heart pepsin digested liquid by PCR, none of them were positive. We do not know the reason for this phenomenon, therefore, more studies investigating this interesting observation should be conducted.

The MAT we used has been extensively employed for the detection of *T. gondii* antibodies in many species, including humans and sheep (Dubey, 2010). Viable *T. gondii* was isolated from 100% (3/3) of sheep with MAT antibodies above 1:800 (Dubey et al., 2014a). The isolation of viable *T. gondii* is the gold standard for detecting live *T. gondii* parasites. However, the success of isolation depends on the tissues tested and the methods used. The density of *T. gondii* cysts in the heart has been shown to be higher than that in the brain or muscle, and the heart is the ideal choice for isolation of *T. gondii* (Dubey et al., 2015). Felid bioassay is the most sensitive method for identification of *T. gondii* (Dubey, 2010). For murine bioassay, the use of immunosuppressed mice facilitates early detection of *T. gondii*. Immunosuppression of mice by dexamethasone has been shown to be a useful method of isolating *T. gondii* (Qian et al., 2012). Moreover, viable ToxoDB#9 strains of *T. gondii* were isolated from cats, pigs, voles and humans from China in a previous study (Dubey et al., 2007; Chen et al., 2011; Wang et al., 2013a,b). Additionally, DNA from *Hipposideros larvatus*, sika deer, goat, *Cebus apella* and masked palm civets was genotyped and identified as ToxoDB#9 (Jiang et al., 2014; Chen et al., 2015; Li et al., 2015; Miao et al., 2015; Cong et al., 2016; Hou et al., 2016). When combined with our results, these findings indicate that ToxoDB#9 is predominant and widespread in animals from China, including sheep. These results indicate the limited genetic diversity of *T. gondii* from China.

The virulence of *T. gondii* was assessed based on their growth rates in cell culture and outbred mice after intraperitoneal injection of dilutions of tachyzoites. The pathogenicity, encystation and growth rate in cell culture of TgSheepHn1 were all stronger than those of TgSheepHn2. ToxoDB#9 isolates were previously reported to have different virulence and pathogenicity in mice (Cheng et al., 2015), which is in accordance with the virulence of the two isolates from sheep observed in the present study. Continued passages of a strain in mice or cell culture can alter the virulence (Dubey, 2010). In the present study, these factors were considered before making conclusions concerning the virulence of strains. Virulence and genome structure analyses of different ToxoDB#9 stains of *T. gondii* showed remarkable variation in ROP 16 and GRA15 (Li et al., 2014). The diversity of ToxoDB #9 stains of *T. gondii* may be connected to the invasion and immune response of this successful parasite.

The results of the present study showed that there is widespread exposure of sheep to *T. gondii* in China. Two viable ToxoDB#9 stains of *T. gondii* were isolated from sheep hearts and found to have different virulence. To the best of our knowledge, this is the first report of isolation of ToxoDB#9 from sheep in China. Because this organism remains present in the tissues of sheep and can therefore infected people via consumption of undercooked meat, sheep pose a risk of *T. gondii* infection and have the potential to impact public health.

## Author contributions

YY performed the data analysis and wrote the manuscript. YF performed the laboratory tests, data analysis. QY participated in the RFLP laboratory test. YW, YL helped in collecting samples. XZ, HL, and LZ helped in the writing of the manuscript.

## Funding

This research project was financed by the Program for Science and Technology Innovation Talents in Universities of Henan Province (Grant No. 17HASTIT038) and China Postdoctoral Science Foundation (2016M600577).

### Conflict of interest statement

The authors declare that the research was conducted in the absence of any commercial or financial relationships that could be construed as a potential conflict of interest.

## Abbreviations

MAT: modified agglutination test
DPI: days post-inoculation
PCR: polymerase chain reaction
ROP: rhoptry protein
GRA: dense granule.

## References

[B1] Bai W. S. Chen Y. Ba L. T. Mai M. T. (2002). Seroprevalence of *Toxoplasma gondii* in domestic animal in Aksu (Sinkiang). Chin. J. Vet. Parasit. 10, 29–30. 10.3969/j.issn.1674-6422.2002.02.011

[B2] Chen C. Y. (2008). Prevalence of *Toxoplasma gondii* in domestic animal in Datong (Qinghai Province). Chin. Qinghai J. Anim. Vet. Sci. 38, 23.

[B3] Chen R. Lin X. Hu L. Chen X. Tang Y. Zhang J.. (2015). Genetic characterization of *Toxoplasma gondii* from zoo wildlife and pet birds in Fujian, China. Iran J. Parasitol. 10, 663–668. 26811736 PMC4724846

[B4] Chen Z. W. Gao J. M. Huo X. X. Wang L. Yu L. Halm-Lai F.. (2011). Genotyping of *Toxoplasma gondii* isolates from cats in different geographic regions of China. Vet. Parasitol. 183, 166–170. 10.1016/j.vetpar.2011.06.013 21757292

[B5] Cheng W. Liu F. Li M. Hu X. Chen H. Pappoe F.. (2015). Variation detection based on next-generation sequencing of type Chinese 1 strains of *Toxoplasma gondii* with different virulence from China. BMC Genomics 16:888. 10.1186/s12864-015-2106-z 26518334 PMC4628340

[B6] Cong W. Qin S. Y. Meng Q. F. Zou F. C. Qian A. D. Zhu X. Q. (2016). Molecular detection and genetic characterization of *Toxoplasma gondii* infection in sika deer (*Cervus nippon*) in China. Infect. Genet. Evol. 39, 9–11. 10.1016/j.meegid.2016.01.005 26772153

[B7] Cui P. Fang S. F. Wu Z. Y. Wu B. Q. (2004). Epidemiological investigation of Toxoplasmosis in farm animals of Hebei Province. Chin. J. Vet. Sci. Technol. 34, 33–34.

[B8] Dong Y. S. Luo Z. Q. Zhang G. C. Liu X. Q. (2011). Prevalence of Toxoplasmosis in sheep and cattle in Qinghai Province. Chin. J. Zoonoses. 27, 359–363. 10.3969/j.issn.1002-2694.2011.04.023

[B9] Dubey J. P. (2010). Toxoplasmosis of Animals and Humans, 2nd Edn Boca Raton, FL: CRC Press; Taylor & Francis Group.

[B10] Dubey J. P. Casey S. J. Zajac A. M. Wildeus S. A. Lindsay D. S. Verma S. K.. (2014a). Isolation and genetic characterization of *Toxoplasma gondii* from alpaca (*Vicugna pacos*) and sheep (*Ovis aries*). J. Trop. Anim. Health 46, 1503–1507. 10.1007/s11250-014-0652-z 25096055

[B11] Dubey J. P. Desmonts G. (1987). Serological responses of equids fed *Toxoplasma gondii* oocysts. Equine Vet. J. 19, 337–339. 10.1111/j.2042-3306.1987.tb01426.x 3622463

[B12] Dubey J. P. Ferreira L. R. Martins J. McLeod R. (2012). Oral oocyst-induced mouse model of toxoplasmosis: effect of infection with *Toxoplasma gondii* strains of different genotypes, dose, and mouse strains (transgenic, out-bred, in-bred) on pathogenesis and mortality. Parasitology 139, 1–13. 10.1017/S0031182011001673 22078010 PMC3683600

[B13] Dubey J. P. Lehmann T. Lautner F. Kwok O. C. Gamble H. R. (2015). Toxoplasmosis in sentinel chickens (*Gallus domesticus*) in New England farms: seroconversion, distribution of tissue cysts in brain, heart, and skeletal muscle by bioassay in mice and cats. Vet. Parasitol. 214, 55–58. 10.1016/j.vetpar.2015.09.004 26391819

[B14] Dubey J. P. Verma S. K. Ferreira L. R. Oliveira S. Cassinelli A. B. Ying Y.. (2014b). Detection and survival of *Toxoplasma gondii* in milk and cheese from experimentally infected goats. J. Food Prot. 77, 1747–1753. 10.4315/0362-028X.JFP-14-167 25285492

[B15] Dubey J. P. Zhu X. Q. Sundar N. Zhang H. Kwok O. C. Su C. (2007). Genetic and biologic characterization of *Toxoplasma gondii* isolates of cats from China. Vet. Parasitol. 145, 352–356. 10.1016/j.vetpar.2006.12.016 17267132

[B16] Feng Y. J. Lu Y. Y. Wang Y. H. Liu J. Zhang L. X. Yang Y. R. (2016). *Toxoplasma gondii* and Neospora caninum in free-range chickens in Henan Province of China. Biomed. Res. Int. 2016:8290536. 10.1155/2016/8290536 27274992 PMC4871953

[B17] Fu Y. J. (2003). Seroprevalence of *Toxoplasma gondii* in swine, cattle and sheep in Qinghai Province. Chin. J. Vet. Sci. Technol. 33, 68–69.

[B18] Hide G. (2016). Role of vertical transmission of *Toxoplasma gondii* in prevalence of infection. Expert. Rev. Anti. Infect. Ther. 14, 335–344. 10.1586/14787210.2016.1146131 26807498

[B19] Hou G. Y. Zhao J. M. Zhou H. L. Rong G. (2016). Seroprevalence and genetic characterization of *Toxoplasma gondii* in masked palm civet (*Paguma larvata*) in Hainan province, tropical China. Acta Trop. 162, 103–106. 10.1016/j.actatropica.2016.06.011 27311389

[B20] Innes E. A. Bartley P. M. Buxton D. Katzer F. (2009). Ovine toxoplasmosis. Parasitology 136, 1887–1894. 10.1017/S0031182009991636 19995468

[B21] Jiang H. H. Qin S. Y. Wang W. He B. Hu T. S. Wu J. M.. (2014). Prevalence and genetic characterization of *Toxoplasma gondii* infection in bats in southern China. Vet. Parasitol. 203, 318–321. 10.1016/j.vetpar.2014.04.016 24813744

[B22] Li M. Mo X. W. Wang L. Chen H. Luo Q. L. Wen H. Q.. (2014). Phylogeny and virulence divergency analyses of *Toxoplasma gondii* isolates from China. Parasit. Vect. 7:133. 10.1186/1756-3305-7-133 24678633 PMC3986613

[B23] Li W. C. (2012). Seroprevalence of *Toxoplasma gondii*, Chlamydia, Brucella in Tibetan sheep. Chin. J. Vet. Med. 48, 58–59.

[B24] Li Y. N. Nie X. Peng Q. Y. Mu X. Q. Zhang M. Tian M. Y.. (2015). Seroprevalence and genotype of *Toxoplasma gondii* in pigs, dogs and cats from Guizhou province, Southwest China. Parasit. Vect. 8, 214. 10.1186/s13071-015-0809-2 25889417 PMC4394553

[B25] Liu Q. Ma R. Zhao Q. Shang L. Cao J. Wang X.. (2010). Seroprevalence of *Toxoplasma gondii* infection in Tibetan sheep in Northwestern China. J. Parasitol. 96, 1222–1223. 10.1645/GE-2601.1 21158639

[B26] Liu Z. K. Li J. Y. Pan H. (2015). Seroprevalence and risk factors of *Toxoplasma gondii* and *Neospora caninum* infections in small ruminants in China. Prev. Vet. Med. 118, 488–492. 10.1016/j.prevetmed.2014.12.017 25591976

[B27] Luo C. Q. Yuan Y. Huang J. M. Huang C. Q. (2013). Epidemic investigation of *Toxoplasma gondii* infection in cattle and sheep in part of Longyan city. Chin. Anim. Health 15, 13–16.

[B28] Miao Q. Huang S. Y. Qin S. Y. Yu X. Yang Y. Yang J. F.. (2015). Genetic characterization of *Toxoplasma gondii* in Yunnan black goats (*Capra hircus*) in southwest China by PCR-RFLP. Parasit. Vect. 8, 57. 10.1186/s13071-015-0673-0 25622613 PMC4316756

[B29] Qian W. Wang H. Su C. Shan D. Cui X. Yang N.. (2012). Isolation and characterization of *Toxoplasma gondii* strains from stray cats revealed a single genotype in Beijing, China. Vet. Parasitol. 187, 408–413. 10.1016/j.vetpar.2012.01.026 22326429

[B30] Su C. Shwab E. K. Zhou P. Zhu X. Q. Dubey J. P. (2010). Moving towards an integrated approach to molecular detection and identification of *Toxoplasma gondii*. Parasitology 137, 1–11. 10.1017/S0031182009991065 19765337

[B31] Wang C. M. He H. X. Qin J. H. Yao S. X. Wang L. R. Liu L. Y. (2005). Investigation of toxoplasmosis in swine and sheep in Xinxiang city Henan Province. Chin. J. Parasit. Parasitic Dis. 23, 31.

[B32] Wang D. Liu Y. Jiang T. Zhang G. Yuan G. He J.. (2016). Seroprevalence and genotypes of *Toxoplasma gondii* isolated from pigs intended for human consumption in Liaoning province, northeastern China. Parasit. Vectors. 9, 248. 10.1186/s13071-016-1525-2 27129860 PMC4851807

[B33] Wang L. Cheng H. W. Huang K. Q. Xu Y. H. Li Y. N. Du J.. (2013b). *Toxoplasma gondii* prevalence in food animals and rodents in different regions of China: isolation, genotyping and mouse pathogenicity. Parasit. Vect. 6:273. 10.1186/1756-3305-6-273 24330536 PMC3849108

[B34] Wang L. Chen H. Liu D. Huo X. Gao J. Song X.. (2013a). Genotypes and mouse virulence of *Toxoplasma gondii* isolates from animals and humans in China. PLoS ONE 8:e53483. 10.1371/journal.pone.0053483 23308233 PMC3538538

[B35] Wang Q. Q. Huang Y. J. Zhang W. Q. Sun H. Chen Y. Kuerbanni T. (2016). Serological survey of toxoplasmosis of sheep from Xinjiang nan jiang farm. Anim. Husb. Feed Sci. 37, 107–108.

[B36] Wang W. L. Zhang Y. X. Zhang F. J. Zhao S. S. Chen Y. X. (2006). Prevalence of *Toxoplasma gondii* in sheep in Beijing. Chin. J. Vet. Med. 42, 29–30. 10.3969/j.issn.0529-6005.2006.09.016

[B37] Wang X. Y. (2007). Seroprevalence of *Toxoplasma gondii* in sheep in Haiyan (Qinghai Province). Chin. Qinghai J. Anim. Vet. Sci. 37, 29.

[B38] Williams R. H. Morley E. K. Hughes J. M. Duncanson P. Terry R. S. Smith J. E.. (2005). High levels of congenital transmission of *Toxoplasma gondii* in longitudinal and cross-sectional studies on sheep farms provides evidence of vertical transmission in ovine hosts. Parasitology 130, 301–307. 10.1017/s0031182004006614 15796013

[B39] Wu S. M. Danba C. Huang S. Y. Zhang D. L. Chen J. Gong G.. (2011). Seroprevalence of *Toxoplasma gondii* infection in Tibetan sheep in Tibet, China. J. Parasitol. 97, 1188–1189. 10.1645/GE-2912.1 21787216

[B40] Yang N. Li H. He J. Mu M. Yang S. (2013). Seroprevalence of *Toxoplasma gondii* infection in domestic sheep in Liaoning Province, Northeastern China. J. Parasitol. 99, 174–175. 10.1645/GE-3201.1 22765359

[B41] Yang Y. Ying Y. Verma S. K. Cassinelli A. B. Kwok O. C. Liang H.. (2015). Isolation and genetic characterization of viable *Toxoplasma gondii* from tissues and feces of cats from the central region of China. Vet. Parasitol. 211, 283–288. 10.1016/j.vetpar.2015.05.006 26033402

[B42] Ye Y. M. Wei D. Q. Tu Y. F. (2002). Seroepidemiological survey of toxoplasmosis in animals from Yunan Province. Chin. J. Parasit. Parasitic Dis. 20, 255.

[B43] Yin M. Y. Wang J. L. Huang S. Y. Qin S. Y. Zhou D. H. Liu G. X.. (2015). Seroprevalence and risk factors of *Toxoplasma gondii* in Tibetan Sheep in Gansu Province, Northwestern China. BMC Vet. Res. 11:358. 10.1186/s12917-015-0358-0 25889907 PMC4345026

[B44] Yuan W. Y. Ma K. Liu C. Y. (2004). Seroepidemiological survey of toxoplasmosis in animals from Heibei Province. J. Med. Pest Control. 20, 80–82. 10.3969/j.issn.1003-6245.2004.02.011

[B45] Yuan Y. H. Ma L. Q. (2007). Seroprevalence of *Toxoplasma gondii* in small tail han sheep in Qinghai Province. Anim. Husb. Vet. Med. 39, 75 10.3969/j.issn.0529-5130.2007.09.031

[B46] Zhang N. Wang S. Wang D. Li C. Zhang Z. Yao Z.. (2016). Seroprevalence of *Toxoplasma gondii* infection and risk factors in domestic sheep in Henan province, central China. Parasite 23, 53. 10.1051/parasite/2016064 27882868 PMC5134671

[B47] Zhang X. Q. Li W. C. Niu X. Y. (2009a). Seroprevalence of *Toxoplasma gondii* in Tibetan sheep in Tianjun Qinghai Province. Chin. Qinghai J. Anim. Vet. Sci. 39, 29 10.3969/j.issn.1003-7950.2009.01.014

[B48] Zhang X. Q. Lu Y. Li W. C. (2009b). Seroprevalence of *Toxoplasma gondii* in Tibetan sheep in Tianjun Qinghai Province. Chin. J. Anim. Health Inspection. 29, 44–45. 10.3969/j.issn.1005-944X.2009.07.022

[B49] Zhang Y.-f. Liu F.-y. Xu X.-p. He L.-x. Li A.-q. Zhang X.-s. (2009). Toxoplasmosis serological test at Northern regions in Xinjiang. Grass Feeding Livestock 4, 22–24.

[B50] Zhao Y. Q. Zhen T. M. Wang J. X. Fu B. Han G. D. (2001). Seroepidemiological survey of toxoplasmosis in animals from Shandong Province. J. Prev. Med. 17, 185 10.3969/j.issn.1006-4028.2001.03.041

[B51] Zhou P. Zhang H. Lin R.-Q. Zhang D.-L. Song H.-Q. Su C.. (2009). Genetic characterization of *Toxoplasma gondii* isolates from China. Parasitol. Int. 58, 193–195. 10.1016/j.parint.2009.01.006 19567233

[B52] Zhu J. F. Chai J. T. Cao Z. G. Wang H. X. Gao M. C. Zhang S. Z. (2007). Seroepidemiological survey of toxoplasmosis in sheep from Henan Province, China. J. Henan Agr. Sci. 6, 124–125. 10.3969/j.issn.1004-3268.2007.06.037

